# Whose voice counts? Family caregivers’ roles in shared decision making for chronic illness in urban South Korea: A qualitative study

**DOI:** 10.1371/journal.pone.0353308

**Published:** 2026-08-06

**Authors:** Go Eun Bae, Jinyoung Lee, Sang-Ho Yoo, Sou Hyun Jang

**Affiliations:** 1 Department of Medical Humanities and Ethics, College of Medicine, Hanyang University, Seoul, Republic of Korea; 2 Department of Sociology, Korea University, Seoul, Republic of Korea; Universitair Kinderziekenhuis Koningin Fabiola: Hopital Universitaire des Enfants Reine Fabiola, BELGIUM

## Abstract

**Background:**

Shared decision making (SDM) is fundamental to patient-centered care, yet dominant frameworks have been developed within dyadic patient–provider models grounded in Western individualistic assumptions. Prior research from East Asian, Sub-Saharan African, and Latin American contexts has documented that medical decision making is frequently family-centered and that caregivers function as recognized actors in clinical encounters. Building on this evidence base, this study examines how caregiver participation is enacted and constrained within the Korean chronic care context, contributing a multilevel reinterpretation rather than a discovery of caregiver involvement.

**Methods:**

We conducted four semi-structured focus group interviews between June and September 2024 with 20 family caregivers of patients diagnosed with hypertension, diabetes mellitus, chronic kidney disease requiring dialysis, or heart disease. Disease-specific groups were formed to enable contextually grounded discussion. Participants were recruited through a Seoul-based research agency and were, on average, highly educated. Using inductive qualitative content analysis, we identified key themes and interpreted the findings through the Social-Ecological Model. Interviews were conducted in Korean, and selected quotations were translated by bilingual researchers through consensus-based reconciliation. Analytic adequacy was assessed in terms of thematic sufficiency.

**Results:**

At the individual level, caregivers experienced emotional strain and decision-related uncertainty, prompting self-directed information seeking. At the interpersonal level, participation was shaped by hierarchical communication with physicians and role-based constraints with nonphysician staff. At the organizational level, fragmented care pathways and standardized information formats constrained access to guidance. At the societal level, culturally embedded deference to medical authority and normalized expectations of family responsibility framed involvement as obligation rather than a formally recognized role. Caregivers simultaneously described aspirations for participatory legitimacy.

**Conclusion:**

Consistent with prior non-Western literature, caregiver involvement is not exceptional but routine; the contribution lies in jointly examining the individual, interpersonal, organizational, and societal conditions through which involvement is simultaneously expected and constrained.

## Background

Chronic illnesses such as hypertension, diabetes mellitus, chronic kidney disease requiring dialysis, and cardiovascular diseases are not characterized by isolated episodes of treatment but instead require long-term management involving continuous monitoring, repeated clinical encounters, and ongoing treatment adjustments [1–3]. Patients and their families are therefore repeatedly confronted with complex medical decisions, including when to initiate or modify pharmacological therapies, whether to pursue invasive interventions, and how to balance clinical effectiveness with quality-of-life considerations [3,4]. Consequently, medical decision making in chronic illnesses is embedded within long-term care trajectories characterized by sustained interaction with healthcare systems rather than discrete, one-time choices [5].

Family caregivers play a central role in these trajectories. Prior research demonstrates that caregivers contribute not only to daily care activities but also to clinical interactions, particularly in the management of chronic and multimorbid conditions [6–8]. In East Asian contexts, where Confucian traditions emphasize filial piety, interdependence, and family responsibility, caregivers commonly function as intermediaries between patients and healthcare professionals [9–12]. Their involvement often includes interpreting medical information, coordinating care, and supporting patients’ decision making, particularly when patients are older or experience constraints in direct participation in clinical communication [13–15]. In such settings, medical decision making frequently occurs not within a dyadic clinician–patient relationship, but through interactions among clinicians, patients, and family members [16, 17].

Despite this relational complexity, much of the existing literature on shared decision making (SDM) has conceptualized decision making primarily at the individual level. SDM has been widely promoted as a cornerstone of patient-centered care, with dominant frameworks emphasizing patient autonomy, health literacy, patient activation, and direct patient–provider communication [18–20]. Influential models, including the SDM conceptualization proposed by Charles et al. and the three-talk framework proposed by Elwyn et al., have made important contributions by framing decision making as an interactive and deliberative process rather than a unidirectional transfer of information [21,22]. These approaches have been instrumental in advancing SDM in collaborative clinical practices.

At the same time, a substantial body of research conducted in non-Western settings has, for more than two decades, documented that medical decision making is often relationally organized and culturally patterned, with family members playing important roles in supporting, mediating, or sometimes constraining patient participation. Studies from East Asian, Sub-Saharan African, and Latin American settings have shown that family involvement in clinical communication and decision making processes is neither peripheral nor exceptional but constitutes a normative feature of care in many healthcare systems worldwide [5,12,23,24,28]. The construct of family involvement in decision making, the proxy and intermediary functions performed by caregivers, and the role of culturally patterned deference have all been articulated in the international SDM literature. Recent reviews further synthesize how relational autonomy, family-mediated information exchange, and institutionally embedded caregiving arrangements operate across diverse health systems [12,27,28]. The present study therefore does not claim that caregiver involvement itself is a novel phenomenon. Instead, it builds on this established evidence base and asks a more specific question: through what structural, institutional, and sociocultural mechanisms is caregiver involvement simultaneously expected and constrained within the particular architecture of Korean chronic care? Three observations motivate this framing. First, although the importance of family involvement in SDM is broadly acknowledged, much of the empirical literature treats the caregiver as a supportive adjunct to a dyadic patient–provider encounter rather than as a meso-level actor whose participation is shaped by its own set of contextual conditions. Second, multilevel analyses that simultaneously examine individual, interpersonal, organizational, and societal conditions of caregiver participation remain comparatively rare, with most studies foregrounding either communicative interaction or cultural values in isolation. Third, the Korean chronic care context—marked by structurally constrained outpatient consultation times, a high concentration of patient volume at tertiary hospitals, and enduring sociocultural expectations regarding family responsibility—has not been systematically connected to the question of how caregiver involvement is enacted in everyday practice. Therefore, the present study seeks to offer a multilevel reinterpretation of how caregiver participation is enacted under these structural, institutional, and normative conditions.

However, these dominant SDM models largely rely on the assumption that participation in decision making can be sufficiently explained through individual-level factors, such as cognitive capacity, communicative competence, and expressed preferences of the patients. These individual-level approaches typically center on the cognitive and communicative capacities of patients while giving limited attention to how illness perceptions, socioeconomic position, educational background, and material constraints shape opportunities for participation in decision making. Although analytically valuable, this focus offers limited explanatory power in contexts where medical decision making is shaped by enduring family relationships, informal caregiving arrangements, and broader sociocultural norms [25–27]. Consequently, the applicability of dominant SDM frameworks to family-centered care environments, where caregiving responsibilities and information interpretation are distributed across multiple actors, remains insufficiently examined in structural rather than cultural terms [12,28].

This research gap is particularly salient in South Korea (hereinafter Korea), where healthcare communication is shaped by Confucian cultural norms, hierarchical clinical authority, and collectivist orientations [9,29]. Family involvement is a routine feature of healthcare delivery. National statistics indicate that 48.8% of hospitalized patients in Korea receive care from family members, with the proportion increasing to 59.1% among patients aged 60 years and older [30]. Despite their substantial involvement, the role of caregivers is rarely formally defined or institutionally supported and is often enacted implicitly within the sociocultural expectations of familial sacrifice [10,31].

The growing burden of chronic diseases has intensified these challenges. In Korea, chronic conditions are projected to account for approximately 80% of all deaths and for most of total healthcare spending by 2024 [32]. Moreover, healthcare expenditures among adults aged 65 years and older have reached more than twice the national average, indicating a concentration of long-term care demands among older populations [33]. Under these conditions, medical decision making is increasingly constituted as an ongoing process embedded within sustained care [2,34].

To address the question of how caregiver involvement is structurally produced in this context, the present study explored how family caregivers of patients with chronic illnesses in Korea experience and interpret their roles. To capture multilevel influences, this qualitative study applied the Social–Ecological Model (SEM) as an interpretive framework [35,36]. This approach enables the positioning of caregiver experiences across individual, interpersonal, organizational, and societal levels, informing the development of culturally attuned SDM models appropriate to the Korean healthcare context [27,37]. The analysis is offered not as a discovery of caregiver involvement but as a structural reinterpretation of how such involvement is enacted under specific institutional and normative conditions.

## Methods

### Study design

This study employed a qualitative descriptive design using focus group interviews (FGIs) to explore the experiences of family caregivers and their perceptions of medical decision making in the context of chronic illness. FGIs were selected to elicit interactive, group-level accounts that could reveal shared concerns and implicit norms that may remain less visible in individual interviews [38,39]. FGI methodology was particularly appropriate given the study's focus on caregivers occupying structurally similar positions within Korea's family-centered care system, as group interaction was expected to surface collective interpretive frames regarding hierarchical authority, family responsibility, and institutional navigation that individual interviews might leave implicit. Following the inductive analysis, SEM was applied as a post hoc interpretive lens to contextualize emergent themes across individual, interpersonal, organizational, and societal levels.

### Study setting and participants

The study was conducted between June 1 and September 24, 2024, at a private interview facility operated by CaseStat Research in Korea. Participants were recruited through purposive sampling facilitated by a research agency, and all interviews were conducted in person at a private facility in Seoul, Korea. The eligibility criteria included being a primary family caregiver of a patient diagnosed with one of the following chronic conditions (i.e., hypertension, diabetes mellitus, chronic kidney disease requiring dialysis, or heart disease) for at least 12 months. Additional inclusion criteria required participants to be between 18 and 80 years of age, fluent in Korean, and actively engaged in caregiving responsibilities. To ensure sustained and direct involvement in clinical care, participants were required to have accompanied the patient to at least four outpatient or inpatient visits within the past year, thereby demonstrating their exposure to clinical consultations and care-related decision making processes. Caregivers with communication difficulties or cognitive impairments were excluded. Twenty caregivers (5 per disease group) were enrolled. Written informed consent was obtained from all the participants. Participants received an honorarium in compensation for their time and transportation expenses, which were reported in accordance with the Consolidated Criteria for Reporting Qualitative Research (COREQ) checklist.

The four-condition, disease-homogeneous design was adopted for three reasons. First, grouping caregivers by patient diagnosis was expected to facilitate contextually grounded discussion by enabling participants to draw on shared caregiving experiences within similar clinical trajectories [38,39]. Second, the four conditions were selected to represent the most prevalent chronic disease burden in Korea, spanning the spectrum from routinely managed conditions (hypertension, diabetes mellitus) to those with higher care intensity and clinical complexity (chronic kidney disease requiring dialysis, heart disease). Together, these conditions account for a substantial share of long-term care demand and exhibit distinct decision making contexts (e.g., the immediacy of dialysis-related decisions versus the longitudinal management of hypertension). Third, the study aimed not to compare disease-specific SDM dynamics but to examine overarching multilevel structural and cultural conditions shaping caregiver involvement; accordingly, the analysis did not treat disease category as an explanatory variable. Cross-condition heterogeneity was addressed analytically by retaining disease group as a contextual identifier throughout coding and by examining, during the post hoc SEM organization stage, whether emergent themes recurred across or were specific to particular conditions. Disease-specific nuances that were not fully exhausted within a single five-person group are acknowledged in the Limitations section as a boundary on thematic sufficiency.

Table 1 summarizes the demographic and caregiving-related characteristics of the 20 family caregivers who participated in FGIs. The mean age of the participants was 43.3 years (standard deviation [SD], 10.59). Approximately 60% were married and slightly more than half (55%) reported a monthly household income exceeding USD 3,600. This income level is above the national median monthly income of wage earners in Korea in 2023 and falls within the upper range of the middle-income bracket based on official wage income statistics [40]. The threshold was used as a contextual reference point to describe the wide range of socioeconomic backgrounds of caregivers, rather than as a policy-based or normative cutoff. The participants demonstrated notably high educational attainment, with 80% holding a bachelor’s degree and 15% holding a graduate degree. Although 45% of caregivers did not cohabit with patients, many reported accompanying patients across primary, secondary, and tertiary care settings.

**Table 1:** Demographic and caregiving-related characteristics of study participants (n = 20).

Characteristic	Total participants (n (%), or mean ± standard deviation)
**Age (years)**	43.3 ± 10.59
**Sex**	
Male	7 (35)
Female	13 (65)
**Household income (monthly)**	
≤ USD 2,500	6 (30)
USD 2,500–3,600	3 (15)
≥ USD 3,600	11 (55)
**Marital status**	
Married	12 (60)
Not married	8 (40)
**Educational attainment**	
Below Bachelor’s degree	1 (5)
Bachelor’s degree	16 (80)
Graduate degree	3 (15)
**Cohabiting with patient**	
Yes	6 (30)
No	9 (45)
Missing response	5 (25)
**Types of hospital visits†**	
Primary hospital	11 (37)
Secondary hospital	10 (33)
Tertiary hospital	9 (30)

**Notes.** Missing responses indicate instances in which specific information was not reported during the interviews.

^†^Multiple selections were allowed. The types of hospital visit describing the range of healthcare settings attended by the participants are presented. They were not used as analytical variables.

Recruitment through a Seoul-based research agency, combined with the high educational profile of the sample, may have produced a participant pool more accustomed to navigating institutional procedures, accessing online and written health information, and articulating expectations regarding clinical communication than the broader Korean caregiving population. Findings should therefore be interpreted as reflecting the experiences of a relatively educated, urban Korean caregiver group rather than as statistically representative of all family caregivers in Korea; the implications of this sampling profile are elaborated in the Limitations.

Table 2 provides individual-level demographic information for each participant, including age, sex, relationship to the patient, and primary diagnosis of the care recipient. It is presented to describe the composition of the study sample and caregiving contexts across disease groups. Given the qualitative and exploratory nature of the study, these characteristics were presented not for subgroup comparison but to describe the study sample and caregiving contexts.

**Table 2:** Individual-level demographic characteristics and caregiving contexts of study participants.

Participant ID	Sex	Age (years)	Relationship to the patient	Primary diagnosis of care recipient
P1	Male	57	Son	Hypertension
P2	Female	51	Daughter	Hypertension
P3	Female	30	Daughter	Hypertension
P4	Male	31	Son	Hypertension
P5	Male	31	Son	Hypertension
P6	Female	44	Spouse	Diabetes mellitus
P7	Female	59	Daughter	Diabetes mellitus
P8	Male	44	Son	Diabetes mellitus
P9	Female	33	Daughter	Diabetes mellitus
P10	Male	49	Son	Diabetes mellitus
P11	Female	34	Granddaughter	Chronic kidney disease requiring dialysis
P12	Female	37	Daughter	Chronic kidney disease requiring dialysis
P13	Male	56	Son	Chronic kidney disease requiring dialysis
P14	Female	41	Daughter	Chronic kidney disease requiring dialysis
P15	Female	43	Spouse	Chronic kidney disease requiring dialysis
P16	Female	33	Daughter	Heart disease
P17	Female	41	Daughter	Heart disease
P18	Female	52	Daughter	Heart disease
P19	Male	65	Son	Heart disease
P20	Female	35	Daughter	Heart disease

**Note.** Participants are identified using anonymized codes. Diagnostic categories refer to the primary chronic condition of the care recipient, as described by the participants.

### Translation procedures

All interviews were conducted in Korean. Verbatim transcripts were prepared in Korean, and quotations selected for inclusion in this manuscript were translated into English collaboratively by one of the corresponding authors (SHJ) and the first author (GE), who conducted all interviews. This collaborative process paired SHJ’s linguistic expertise as a bilingual Korean–English speaker with GE’s firsthand contextual knowledge of the interviews, ensuring that the selected quotations were accurately translated while preserving the participants’ original nuances. To further enhance translation accuracy and conceptual consistency, the translated excerpts were independently reviewed through back-translation and reconciliation procedures conducted by SHJ and GE. Discrepancies between the original Korean transcripts, English translations, and back-translated Korean versions were discussed within the research team until consensus was reached. Particular attention was paid to culturally embedded concepts that lack direct English equivalents, including filial responsibility (hyo), hierarchical deference in clinical encounters, and relational expressions of family obligation. Where direct translation risked obscuring contextual meaning, translation decisions were iteratively discussed to preserve semantic, relational, and cultural meanings while maintaining participants’ original voices in the reported quotations.

### Researcher reflexivity

Reflexivity was maintained throughout data collection and analysis. The primary moderator (GE) is a former clinical nurse with 12 years of nursing experience and a PhD in sociology who conducts qualitative research on SDM. Her clinical background provided familiarity with hospital environments, interprofessional communication, and the language used to describe clinical encounters, which likely facilitated rapport with participants and aided interpretation of accounts related to ward-level workflow and healthcare communication. In Korean clinical settings, nurses often occupy intermediary positions between physicians, patients, and family caregivers rather than maintaining relationships of professional equivalence with physicians. This background may therefore have enhanced sensitivity to caregivers’ experiences of coordination, communication barriers, and hierarchical negotiation within healthcare settings. At the same time, prior experience within hierarchical medical institutions carried the possibility that certain organizational norms or clinical interactions could be interpreted as familiar or routine rather than critically interrogated. Participants may also have assumed that the moderator possessed insider knowledge of healthcare systems, potentially influencing how criticism toward clinicians or institutions was expressed. To mitigate these risks, GE adopted a deliberately non-directive facilitation style, used open-ended probes inviting both critical and appreciative accounts, and refrained from introducing evaluative clinical terminology during discussion. The assistant moderator (JY) holds a master’s degree in sociology and was a doctoral student at the time of the study. She was responsible for recording field notes, documenting nonverbal expressions and group dynamics, and observing interactional patterns during the FGIs. Her sociological training contributed to orienting the analytical process toward structural, relational, and normative dimensions of caregiver experience beyond the clinical content of participants’ narratives. The wider research team’s engagement with SDM scholarship, particularly the corresponding authors’ expertise in this field, also carried a potential interpretive risk, including the possibility of privileging autonomy-supportive or participatory interpretations of caregiving experiences. This possibility was addressed through reflexive memo writing after each FGI and coding round, discussion of alternative interpretations during team meetings, and an explicit effort to prioritize participants’ own understandings of appropriate caregiving and decision making roles. Where divergent interpretations emerged among team members, these differences were treated as analytic material rather than resolved solely through consensus.

### Procedure

Before participation, all participants were fully informed, both verbally and in writing, of the purpose, procedures, risks, and protections of the study. Participants were made aware of their right to withdraw from the study at any time without penalties. Identifiable information was anonymized and all data were stored securely in encrypted password-protected files that were accessible only to the research team.

Four FGIs were conducted, one for each chronic disease group, with five caregivers per group. The interviews lasted approximately 90–120 minutes and were conducted by two qualitative researchers (GE and JY). GE, a female researcher and former nurse with a PhD in sociology who conducts qualitative research on shared decision making, served as the primary moderator and facilitated the discussion using a semi-structured interview guide, while JY, a female doctoral student in sociology at the time of the study, acted as the assistant moderator, recording field notes and monitoring group dynamics. Both researchers had prior training and experience in qualitative research and focus group facilitation.

The semi-structured interview guide was initially developed based on existing SDM literature and then refined through several research team meetings involving GE, JY, and SHJ. During this process, the wording, sequence, and thematic scope of the questions were reviewed to ensure that the guide adequately captured caregivers’ roles, communication experiences, and participation in medical decision making. The final version was reviewed and confirmed by SHY and subsequently pilot tested with caregivers who were not included in the main study. The key domains included caregivers’ roles and responsibilities, communication with healthcare providers, participation in medical decision making, emotional burden and coping strategies, unmet needs, and desired forms of support (S1 File).

All interviews were audio recorded and transcribed verbatim. The transcripts were crosschecked with field notes to ensure contextual accuracy. The research team took additional precautions to monitor emotional fatigue or distress among participants and offered referrals to mental health or counseling services as needed. This study was approved by the Institutional Review Board of Korea University (Approval No. KUIRB-2024-0156-04).

### Data analysis

The data were analyzed using an inductive qualitative content analysis approach, which is consistent with conventional qualitative content analysis methods that derive analytical categories from the data rather than from predefined theoretical constructs [41,42]. This approach was used to examine patterns and themes emerging from the participants’ accounts without applying an a priori coding framework, in line with inductive qualitative inquiry traditions [41–43]. Given the FGI design, the analysis considered both individual statements and interactional features of group discussions, including shared meanings developed through participant interactions [38,39].

All interview transcripts were imported into MAXQDA 24 for data management and analysis. Three researchers (GE, SHJ, and JY) independently reviewed the transcripts and conducted open coding to identify meaning units and generate initial codes, following the inductive coding procedures described in grounded theory–informed qualitative analysis [42,43]. A preliminary codebook was developed based on the initial coding results. The codebook was subsequently reviewed and revised through regular team discussions, during which code definitions were clarified, overlapping or ambiguous codes were reorganized, and conceptual distinctions were adjusted, consistent with recommended practices in qualitative content analysis [41,42].

The resulting codebook organized caregiver accounts across major domains, including chronic-illness characteristics, information-seeking behavior, health literacy, physician communication, nurse and allied-staff communication, caregiver communicative positioning, caregiver role, provider information provision, relational dynamics, SDM experience, inter-institutional comparison, organizational information provision, structural features of service delivery, perceptions of ideal SDM, images of physicians and medical culture, and structural limits of the medical system (S2 File).

During the iterative analytical process, the codebook was revised seven times. Revisions were made to refine the code definitions and to improve consistency across the research team. As the analysis progressed, transcripts were redistributed among team members for further coding using the revised codebook while remaining open to the identification of additional codes. After the coding framework reached a stable structure, all transcripts were recoded using the finalized codebook to ensure consistency across datasets [41,42].

Analytic rigor was addressed through iterative team discussions, reflexive memo writing, and consensus building among researchers rather than through statistical measures of intercoder reliability, which are not uniformly required in interpretive qualitative content analysis [41,44].

Thematic sufficiency, also referred to as data adequacy, rather than absolute thematic saturation, was monitored throughout the analytical process. Because each focus group represented a distinct chronic-illness context, the team did not claim saturation across all possible disease-specific nuances. Instead, after each coding round, the team assessed whether additional analysis generated substantively new meaning units beyond minor elaborations of established themes [45,46]. After the fourth focus group, no new major themes emerged at the cross-disease level, and the structural and normative patterns organizing the present findings were stable across groups. Disease-specific nuances that may not have been sufficiently explored within a single five-person group were acknowledged as limitations on the interpretive scope of this study and are discussed in the Limitations section [44,47].

After inductive coding and theme development were completed, the resulting categories were organized post hoc using SEM as an interpretive framework. SEM was used to organize and contextualize findings across individual, interpersonal, organizational, and societal levels rather than as a deductive coding structure or explanatory theory [35,36,48]. This framework provided a structured approach to examine how caregiver experiences in medical decision making were situated within multiple levels of influence while maintaining the inductive orientation of the analysis [41–43].

Concretely, after the codebook stabilized, each major theme was reviewed by the full team and assigned to the SEM level that best reflected the predominant ecological locus of the experience described—while explicitly recognizing that several themes cut across levels (for example, hesitation to question physicians was simultaneously an interpersonal-encounter dynamic and a societal-normative pattern, and caregiver information-seeking spanned individual motivation, interpersonal peer exchange, and organizational information provision). Cross-cutting themes were retained at their primary level but cross-referenced in the Results where this dual location was analytically important. No themes were discarded for poor fit with the framework; in cases where a theme did not map cleanly onto a single SEM level, the team treated this resistance as analytically informative rather than as a problem to be smoothed away.

## Results

### Findings based on the application of the SEM

**Fig 1:**
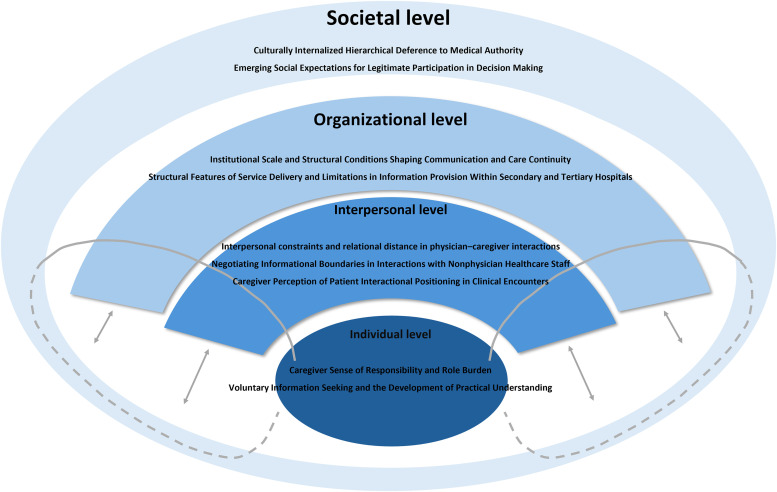
Social-ecological framework of family caregiver involvement in SDM. The model depicts interactions at individual, interpersonal, organizational, and societal levels. Solid arrows indicate direct relationships between levels, whereas dashed arrows represent indirect or normatively mediated influences. Bidirectional arrows reflect the recursive and multidirectional nature of these relationships.

This study applied SEM to integrate and organize the accounts of family caregivers of SDM involvement in patients with chronic illnesses. Fig 1 presents a multilevel framework developed through an inductive thematic analysis, illustrating how caregiver experience and roles in SDM are shaped by dynamics across four interrelated levels: individual, interpersonal, organizational, and societal.

Before turning to the level-specific findings, two interpretive notes apply throughout. First, because the study analyzes caregiver-perspective accounts, findings at the interpersonal, organizational, and societal levels reflect caregivers’ experiences and interpretations of clinical and institutional processes rather than a direct reconstruction of provider behavior, policy intent, or population-level dynamics. Structural claims (e.g., “structural externalization,” “participation paradox”) are advanced as interpretive findings grounded in caregiver narratives and converging across multiple participants, not as empirical characterizations of institutions themselves. Second, while caregivers and patients are analytically distinct, in the family-centered context of this study caregivers frequently described their role as inseparable from the patient's clinical trajectory; this dual positioning is preserved in the accounts below. Additional anonymized excerpts illustrating each level are provided in S3 File.

At the individual level, caregiver narratives reflected a sense of responsibility, role burden, and voluntary information seeking, including the emergence of lay expertise. At the interpersonal level, caregivers described constraints and relational distance in interactions with physicians, unmet informational expectations in encounters with nonphysician healthcare staff, and the ongoing negotiation of their interactional positioning during clinical encounters. At the organizational level, caregivers emphasized differences in communication and continuity between large hospitals and local clinics as well as internal service delivery structures that shaped access to actionable information. At the societal level, the caregivers situated these experiences within the context of broader cultural perceptions of medical authorities and widely shared expectations regarding family responsibility for care coordination.

Rather than functioning as isolated layers, these levels were interconnected and mutually shaped. Caregiver participation in SDM emerged at the intersection of emotional and cognitive demands, interactional conditions, organizational arrangements, and sociocultural norms. Thus, the framework conceptualizes caregiver involvement not as an individual attribute or discrete role but as a product of multilevel and recursive processes embedded within specific clinical and cultural contexts.

### Individual-level: Internalized responsibility and cognitive-emotional burden in SDM

At the individual level, caregiver participation was shaped by uncertainty, emotional strain, and sustained information-seeking efforts. Caregivers gradually developed a practical understanding through repeated engagement with medical information and peer or institutional resources. These efforts reflected not an assertion of decision authority but an internalized sense of responsibility in managing uncertainty within family-centered care.

### Caregivers’ sense of responsibility and role burden

Drawing on data from FGIs, caregivers frequently described experiencing emotional strain and cognitive burden associated with their perceived responsibility in healthcare decision-related situations. They often navigated complex medical information and responded to clinical circumstances, particularly when explanations were insufficient or fragmented. This sense of responsibility was frequently accompanied by self-doubt and anxiety, prompting ongoing reflection and concern about the appropriateness of their judgments. For instance, P11, an unmarried granddaughter caring for her grandmother who was undergoing dialysis, described acute uncertainty and informational vulnerability in an emergency decision making context.

*“My grandmother had kidney issues initially, but the primary diagnosis was a stroke. I knew her kidneys weren’t great, but the focus was on the stroke… (omitted)… I didn’t know that kidney issues meant she shouldn’t drink much water. I thought drinking more water would help with bowel movements because that’s what I saw on TV. But suddenly her abdomen was swelling, and she couldn’t breathe... We ended up in the ER. I was overwhelmed, I didn’t know anything. There wasn’t even a real explanation. They just handed me a consent form and started dialysis right away.” [P11]*

This account illustrates the psychological and emotional challenges that caregivers encountered in high-stakes, time-sensitive situations. Limited or unclear medical explanations contributed to confusion and uncertainty in moments that require rapid decision making. In such contexts, caregivers described assuming practical responsibility for decision-related processes without feeling fully informed or adequately supported.

These experiences suggest that the emotional strain experienced by caregivers was not described solely as individual anxiety but as emerging within circumstances characterized by informational asymmetry and limited institutional guidance. Under these conditions, caregivers experienced a heightened sense of responsibility in supporting or facilitating decision making processes despite not being formally recognized as members of the decision making team. Rather than reflecting an intentional transfer of authority, this involvement appeared to arise from situational demands within a family-centered care context in which caregiver participation was implicitly expected.

### Voluntary information-seeking and the development of practical understanding

The caregivers described engaging in sustained and self-initiated information-seeking practices as part of their efforts to manage the uncertainty and responsibility they experienced in decision-related situations. Although such behavior may initially appear proactive, participants’ accounts indicated that information seeking often emerged in response to perceived vulnerability and the need to minimize the risk of misunderstanding clinical information. This tendency was particularly evident when patients were older, clinically unstable, or less able to actively participate in medical communication.

Within this context, caregivers frequently described feeling that they “had to know” enough to protect the patient’s interests. Therefore, information seeking was framed not primarily as an expression of autonomy or confidence but as a way of reducing anxiety associated with possible misinterpretation or omission during decision making processes. P18, a married woman who regularly accompanied her mother who had heart disease to outpatient follow-up visits and lived with her, articulated this sense of responsibility as follows:

*“Even if the doctor explains something, it doesn’t feel sufficient. If I don’t understand it properly, it could eventually harm my mother. As a caregiver, I feel that I need to understand enough to ask questions; otherwise, my mother might be disadvantaged in the treatment process. So, I try to study what I’m curious about before appointments. When your own family member is in this situation, you become more attached and more anxious. Being ill makes it hard for the patient to think rationally, so I feel I need to know more to compensate for that.” [P18]*

In this context, information seeking is closely intertwined with emotional strain and perceived accountability. The emphasis on “understanding enough” reflects a cognitive burden associated with interpreting medical explanations, whereas the concern about potential harm indicates an underlying anxiety about making inadequate judgments. Rather than claiming authority in decision making, P18 positions herself in an attempt to compensate for what she perceives as her mother’s temporary cognitive or emotional vulnerability.

Other caregivers described different pathways of acquiring knowledge. P15, a woman in her early forties whose husband was undergoing dialysis for chronic kidney disease and whom she accompanied regularly to nephrology visits, emphasized learning through peer-based exchanges. Given the frequency of dialysis sessions, she described spending extended time in hospital waiting areas and online communities as follows:

*“Caregivers wait during dialysis, and they talk to each other and share information. In Korea, Naver cafés are the most well-established… I looked for the largest one, joined it, and just spent the whole day reading. Some symptoms are common, but others are very individual. Doctors explain in general terms, but when something unusual happens, you need to hear from people who have actually experienced it. On dialysis days, I also listened to other patients and caregivers in the waiting area. That is how I figured things out.” [P15]*

Here, peer-based exchanges functioned as supplementary sources of practical knowledge when generalized clinical explanations were insufficiently individualized. The accumulation of experiential information gradually occurred through repeated exposure, suggesting an incremental development of condition-specific understanding rather than an explicit pursuit of expertise.

In contrast, P12, an unmarried woman working as a university researcher whose mother (a former nurse) was undergoing dialysis, described relying heavily on institutional and academic resources.

*“At one point, no hospital would help, so I called the Health Insurance Review and Assessment Service directly… I checked again and again and searched for papers and books myself. I looked up both domestic and international studies. If there were unusual symptoms or questions about support policies, I contacted the relevant institutions directly to confirm.” [P12]*

Although the informational strategies differed, peer networks in one case and formal institutional or academic sources in another, both patterns reflected similar dynamics. Caregivers assumed responsibility for clarifying uncertainties when clinical communication was perceived as limited, generalized, or difficult to translate into patient-specific implications.

In all cases, caregivers did not explicitly identify themselves as possessing professional-level expertise. However, their sustained interaction with medical information, institutional systems, and experiential knowledge networks contributed to the gradual development of practical, experience-based competencies. Voluntary information seeking was closely connected to the emotional strain and cognitive burden described earlier in this section. Rather than representing an expansion of caregiver authority, it reflects how caregivers internalized responsibility under conditions of uncertainty.

### Interpersonal-level: Relational constraint and positioning in SDM

At the interpersonal level, caregiver participation was shaped by three interrelated dynamics: interactional constraints within physician consultations, negotiation of role-based informational boundaries with nonphysician staff, and caregivers’ interpretations of patient relational positioning during clinical encounters.

### Interpersonal constraints and relational distance in physician–Caregiver interactions

At the interpersonal level, the caregivers described the communicative and relational conditions that influenced their engagement with physicians during clinical encounters. These conditions were experienced not only in terms of information exchange but also in how caregivers understood their position within the consultation setting.

Several participants noted that, during the initial phase of diagnosis, physicians tended to provide relatively detailed explanations. However, as care became more routine, consultations became increasingly brief and procedural. This shift was associated with fewer opportunities for clarification and limited space for dialogue. P7, a married woman in her late fifties who lived with her mother and regularly accompanied her for diabetes management, reflected on this change over time:

*“At first, they explained everything in detail, what each term meant, what should be reduced or increased. But now it has been about three or four years since my mother was diagnosed... During regular checkups, there is no explanation. They just say, ‘Nothing unusual, right?’ and it ends like that.” [P7]*

In this account, the transition from a detailed explanation to an abbreviated follow-up was described as a narrowing of interactive exchange. The experience was not framed as a direct exclusion but rather as a gradual reduction in communicative depth that altered expectations about participation.

Caregivers also pointed to the structural features of consultations, such as limited appointment times and high patient volumes, as contextual factors shaping interactional dynamics. In such settings, raising additional questions was difficult. P2, a married woman in her early fifties who regularly accompanied her mother for hypertension management, described the following constraints:

*“When you go into the consultation room, the time is so short. The doctor just talks through their points, and when the patient or caregiver tries to ask something, you get this feeling that they are annoyed…” [P2]*

Here, time pressure was not described as explicit refusal but as creating an atmosphere in which the extended dialogue felt constrained. This perception appeared to indicate how the caregivers decided when and how to speak. Beyond situational constraints, several participants interpreted these encounters within a broader relational framework characterized by asymmetries in medical knowledge and authority. P1, a married man in his late fifties who regularly accompanied his father on follow-up visits for hypertension, articulated this perception:

*“It may seem like a horizontal relationship, but the doctor–patient dynamic is more like a tilted playing field. As I mentioned earlier, the structure of Korea’s medical system produces a hierarchy—where one feels positioned at the bottom and must lower oneself to be heard. … (omitted) … Medical knowledge itself is inherently asymmetrical.” [P1]*

Rather than focusing solely on communication style, P1 emphasized the structural differences in knowledge and authority that shaped interactional expectations. This perspective suggests that caregiver participation was influenced not only by time or explanation patterns but also by how they understood their relative standing within the clinical relationship. Together, these accounts indicate that interpersonal constraints operated across multiple dimensions, including routinized communication over time, situational limits within consultations, and perceived asymmetries in authority. These conditions did not eliminate caregiver involvement; rather, they shaped how caregivers calibrated their engagement, sometimes leading to more reserved or cautious interactions. Such relational distance emerged gradually within ongoing care relationships and may have implications for caregiver perceptions of their roles within SDM processes.

### Negotiating informational boundaries in interactions with nonphysician healthcare staff

During interpersonal encounters with nonphysician healthcare staff, caregivers described repeated efforts to obtain practical and administrative information relevant to the ongoing treatment. These interactions often involved sequential questioning across multiple personnel, rather than a single, continuous exchange. Consequently, the caregivers experienced the process of information seeking as fragmented and interactionally discontinuous. P14, a married woman in her early forties who regularly accompanied her mother to nephrology clinics for chronic kidney disease follow-up, described navigating a series of interactions while preparing for dialysis.

*“To undergo dialysis, my family member had to have surgery to create an artificial blood vessel in the arm, and there were so many things to prepare. They mentioned something called a Special Case Co-payment Reduction Program, but even the term itself was hard to understand. A nurse explained that we needed to apply and complete the registration, but when I asked the outpatient nurse about the costs, they said they did not know and told me to check with the administrative office. When I went to the administrative office, the staff there told me to ask the social work department instead” [P14]*

In this account, the difficulty was not framed as the unwillingness of individual staff members, rather, the interaction unfolded as a chain of referrals across departments, requiring the caregiver to repeatedly restate questions and reposition herself in different communicative settings. The experience was procedurally complex and interactionally segmented, making it unclear where definitive explanations could be obtained. The caregivers also differentiated between relational accessibility and decision-related authority in their interactions. Nurses were frequently described as approachable and responsive to everyday exchanges, particularly in outpatient settings. However, when questions shifted toward prescriptions, costs, or eligibility criteria, caregivers encountered limitations in the scope of information that nurses felt authorized to provide. P10, a man in his late forties who regularly accompanied his mother to endocrinology visits for diabetes management, articulated the following distinction:

*“When I ask nurses, they do explain things kindly. They are always nearby and take care of us. But when it comes to things like prescriptions or costs, it eventually becomes, ‘That is not something I can explain.’ Nurses are not doctors, so they cannot really go into detail about those issues.” [P10]*

Here, the interaction was characterized by a shift in communicative boundaries; while relational support remained available, informational authority appeared to be narrow. This distinction shaped how the caregivers formulated subsequent questions and determined which concerns were appropriate for particular encounters. During the interviews, caregivers described recurring patterns of interactional adjustment. Rather than passively accepting incomplete information, they repeatedly engaged with staff members, reformulated their questions, and navigated role-based limitations in real time. In this context, unmet informational expectations emerged not simply from the absence of information, but from how information was sequentially requested, redirected, and only partially addressed across encounters.

Collectively, these accounts indicate that caregiver experience with nonphysician healthcare staff was shaped by different institutional roles as it became enacted within everyday interactions. Their efforts to obtain coherent guidance unfolded within communicative boundaries structured by professional scope and organizational division, requiring sustained interactional work on the part of the caregivers.

### Caregiver perceptions of patient interactional positioning in clinical encounters

Analysis of caregiver-focused FGIs indicated that caregivers interpreted patients’ participation in consultations not solely in terms of communicative engagement, but in terms of how patients positioned themselves in relation to physicians during clinical encounters. Caregivers described differences in how patients expressed symptoms, responded to recommendations, and negotiated care, and they understood these patterns as relationally shaped within the consultation context. One pattern involved the minimization or withdrawal of symptoms during consultations. P17, a married woman in her early forties who regularly accompanied her mother for heart disease treatment, described how her mother’s symptom reporting changed depending on the context.

*“When she is with me, she says she feels short of breath or that her chest feels tight. … But when we enter the consultation room, she suddenly says she is fine. She tells me that once she is standing in front of the doctor, she cannot remember what she was going to say. … And if she says something hurts, she worries the doctor might suggest more tests. She says it feels like she would be making a bigger issue out of it, so she just says she is okay.” [P17]*

In this account, the consultation room was described as a setting in which symptom disclosure was modified. Discomfort acknowledged in private was reframed as “being fine” in front of the physician. The caregiver associated this shift with concerns about additional examinations and difficulties in articulating perceived problems during the encounter. As a result, the patient was perceived as having limited symptom expression in that context, and the caregiver described restating or supplementing information during the visit. This pattern was understood as a deferential form of interactional positioning in which the patient adjusted disclosure in response to the consultation setting.

A contrasting pattern was described by P7, a married woman in her late fifties who regularly accompanied her mother for diabetes. She characterized her mother’s responses to medical advice as being more direct:

*“When the doctor says she needs to take more medication, my mother says, ‘I do not need that. I can manage it with diet.’ … There is a kind of atmosphere like she is ready to argue. I feel anxious sitting next to her. I wish she would ask more gently. The doctor does not push strongly either—he just speaks vaguely and lets it pass. Then my mother says she does not fully trust him… In the end, I find myself constantly adjusting things between them.” [P7]*

The patient’s position is described as openly questioning or resisting medical recommendations. The caregiver perceived tension in the exchange and assumed a moderating role. Rather than indicating simple nonadherence, this interaction reflected assertive positioning in which the patient actively articulated preferences and evaluations. The caregiver’s account highlights how this positioning shaped the relational atmosphere of the consultation and required ongoing coordination.

The third pattern involved negotiated adjustment. P6, a married woman who regularly accompanied her husband to diabetes follow-up visits, described a consultation in which he expressed a desire to discontinue the medication.

*“My husband told the doctor he wanted to stop taking the medication. The doctor suggested trying it conditionally; if he maintained his weight for three months, they would reconsider. He managed to keep it stable; his numbers went down to 6.5, and he stopped the medication for two years.” [P6]*

In this case, the patient’s preference was acknowledged and incorporated into the conditional plan. The caregiver described this exchange as involving the mutual adjustment, with treatment decisions linked to agreed-upon criteria and monitoring. This interaction was perceived as a collaborative form of positioning, distinct from symptom minimization and open resistance.

Across these accounts, caregivers perceived patients’ participation in clinical encounters as being shaped by how they positioned themselves in relation to physicians, ranging from limiting disclosure to openly challenging recommendations and engaging in negotiated adjustment. These patterns were described as context-dependent responses within specific consultation settings rather than as fixed personal traits. The roles of the caregivers were shaped accordingly as they clarified symptoms, moderated exchanges, or supported negotiated agreements within decision-related interactions.

### Organizational-level: Institutional and structural constraints in SDM

At the organizational level, caregiver engagement was shaped by cross-institutional differences in scale, infrastructure, and internal service configurations, which affected coordination, information delivery, and continuity of care.

### Institutional scale and structural conditions shaped communication and care continuity

At the organizational level, caregivers described how differences in institutional scales, scheduling systems, and resource distribution shaped their experiences of communication and care continuity across healthcare settings. These differences were not attributed solely to individual clinicians but were understood as embedded in broader structural arrangements within tertiary hospitals and local clinics.

Large university-affiliated hospitals were generally perceived as providing specialized expertise and comprehensive diagnostic evaluations. However, caregivers also described these settings as operating within rigid scheduling systems and with high patient volumes, which constrained interactional flexibility. P20, a woman in her mid-thirties who regularly accompanied her mother to outpatient cardiology visits at a university hospital following pacemaker implantation, illustrated how appointment systems and workflow intensity limited opportunities for extended dialogue.

*“At university hospitals, if you try to change an appointment, the default is three months later. Even if there is an open slot in between, it is not easy to adjust. As a caregiver, you worry whether the patient’s condition might worsen during that time. But when you actually go to the hospital, even if you arrive 30 minutes before your scheduled time, you do not get seen right away. Waiting one or two additional hours is normal. And once you finally go in, the doctor just says, ‘Your numbers look fine,’ and it is over in less than a minute… Patients are lined up in the hallway, and the doctor moves back and forth between two rooms. It feels structurally difficult to ask questions.” [P20]*

In this account, limited communicative space emerged from institutional workflow patterns, including extended waiting times, compressed consultations, and visible patient throughput, rather than from interpersonal reluctance alone. In contrast, local clinics were often described as more interactionally accessible. P9, a woman in her early thirties who regularly accompanied her mother for diabetes management, emphasized differences in communicative responsiveness:

*“Local clinics have fewer patients and seem less busy, so they feel more prepared to listen. My mother has diabetes, and I manage her meals, and at the local clinic we even receive text messages about foods to avoid or exercise after meals. It feels like they are more interested in the patient’s daily life. If the caregiver or patient asks questions, they try to answer sincerely. But at university hospitals… it feels like the whole structure is designed to process more patients mechanically.” [P9]*

A smaller patient volume and individualized follow-up were associated with greater accessibility and attentiveness to daily disease management. However, caregivers did not perceive local clinics to be uniformly preferred. P19, a married man in his mid-sixties who accompanied his mother for cardiac follow-up, described a structured transition from a university hospital to a local clinic after her condition had stabilized. His mother previously received multidisciplinary care for cardiac disease, hypertension, and diabetes at a tertiary hospital, where she underwent regular diagnostic testing. After surgical intervention and clinical stabilization, she was formally referred to a local clinic for routine management, with an explanation that she would return to the university hospital for biannual follow-up. Reflecting on this shift, he stated:

*“My mother had diabetes and hypertension and was seeing specialists at a university hospital… They did regular tests there. But after her surgery, the doctor said her condition was stable and that she did not need to come as often, so we were referred to a local clinic. At the clinic, they look through the documents from the hospital and prescribe medication every two months. I want more detailed information. Of course, the doctor is kind, and the nurses are different from the dry tone at the university hospital. But objectively speaking, local clinics do not have many diagnostic devices beyond basic X-rays. I wonder, is she really fine? Or could something be missed?” [P19]*

In this account, the referral-based redistribution of care responsibilities was not described as inappropriate but was accompanied by uncertainty regarding monitoring depth. Although relational accessibility increased in local clinics, perceived technical comprehensiveness was comparatively limited. Across the interviews, organizational-level differences were observed in scheduling rigidity, consultation duration, patient volume, referral pathways, and diagnostic infrastructure. These structural contrasts shaped the caregiver experience with communicative space and care continuity and influenced how they navigated decision-related processes across institutional contexts.

### Structural features of service delivery and limitations in information provision within secondary and tertiary hospitals

Although the previous subtheme examined structural contrasts across institutional settings, this subtheme focuses on internal service configurations within secondary and tertiary hospitals. Caregivers described how care processes were organized within large hospital systems and how these internal arrangements shaped access to information, coordination across departments, and practical guidance during treatment.

Participants frequently noted that the clinical pathways within general and tertiary hospitals were segmented across departments, diagnostic units, and administrative offices. Even when care was delivered at a single institution, laboratory testing, imaging services, outpatient consultations, and procedural preparations were often distributed across separate units. Caregivers assumed the responsibility for tracking documentation, confirming procedural steps, and reconciling recommendations across departments, particularly in long-term or complex treatment trajectories.

Beyond coordination challenges, caregivers emphasized the limitations in how information was structured and delivered within hospital systems. Guidance was often provided through standardized written materials with limited contextual explanation or adaptation to patient-specific circumstances. P13, a married man in his mid-fifties who accompanied his mother for dialysis-related nephrology visits approximately every two months, described the informational challenges associated with dietary management for dialysis patients:

*“For dialysis patients, there are actually more foods they cannot eat than foods they can. So, as caregivers, we have no choice but to focus heavily on diet management. What patients eat directly affects their condition and whether they can even undergo dialysis properly… But when you look at the leaflet from the hospital, it is just a long list of foods not to eat. It does not explain what is actually possible, or how to apply it in daily life. There is no contact point for asking how this applies to a specific patient. It feels like a leaflet for display. I get the sense they hand out the same material every year.” [P13]*

In this account, the issue was not the absence of informational materials, but the standardized format and limited interpretive support accompanying them. Written leaflets emphasized restrictions without any corresponding practical guidance or institutionalized channels for clarification. Consequently, caregivers independently translated the generalized instructions into patient-specific decisions.

By contrast, a small number of participants described hospital contexts in which informational support was formally integrated into organizational routines. P18, a woman in her early fifties whose mother was hospitalized for heart valve replacement surgery, recounted her experience in a hospital with a dedicated cardiac center and a structured caregiver education system. In addition to routine consultations with the attending physician, designated staff members were assigned to provide procedural explanations during scheduled sessions.

*“Before the surgery, caregivers were gathered separately in a seminar room. They showed us materials about how the surgery would be performed and explained everything step by step. There was a dedicated staff member responsible for providing this information not just the attending physician. After the surgery, we also received separate guidance on postoperative care. They called it caregiver education, and it was conducted twice, each session lasting about an hour. I felt the hospital had a well-organized educational system in place.” [P18]*

The informational provision was formally structured and embedded in the organizational design of the hospital. Caregivers emphasized that such coordinated educational processes were department specific and not consistently established across chronic care settings. Across the interviews, access to meaningful guidance within secondary and tertiary hospitals for caregivers was shaped by internal service configurations, including departmental segmentation, distributed procedural responsibilities, and standardized information formats. Variability in how hospitals embedded coordination and educational mechanisms into routine practice influenced caregiver clarity and confidence in navigating treatment-related decisions, underscoring the importance of organizational dimensions of SDM support within large hospital systems.

### Societal-level: Medical authority, hierarchical norms, and shifting expectations in SDM

At the societal level, caregiver participation reflected the coexistence of culturally internalized hierarchical deference to medical authorities and emerging expectations for socially legitimate participation in decision making.

### Culturally internalized hierarchical deference to medical authority

At the societal level, caregivers’ accounts reflected socially shared norms regarding the authority of physicians. Medical authority was considered not merely as professional expertise but also as a culturally patterned hierarchy that shaped how patients and caregivers positioned themselves before entering the consultation room. Physicians were widely perceived as occupying a high-status role in Korean society. This perception appears to operate as an assumption that medical judgment is inherently superior and should not be questioned easily. P5, a man in his early thirties who regularly accompanied his father for hypertension management, described how this hierarchical orientation was learned and normalized through broader socialization processes:

*“Basically, older people tend to comply unconditionally with what the doctor says. In Korean society, the social status of doctors is extremely high. They are selected from the most outstanding people, so there is a strong belief that whatever they say must be correct. I think that has become culturally internalized. It is not something people consciously decide—it just feels natural. … Even for me, raising a counterargument feels uncomfortable. Even if the doctor is not intentionally authoritative, I still feel intimidated. I think, ‘The doctor probably knows more than I do. Of course, the doctor must be right.’ And then I hold myself back.” [P5]*

In this account, hesitation was framed as being socially learned and normalized. P5 emphasized that even as a younger caregiver, he experienced difficulty articulating counterarguments, suggesting that hierarchical positioning may operate independently of age or educational background. A similar orientation was described by P16, an unmarried woman in her early thirties who regularly accompanied her mother for cardiac disease follow-up. Although she did not live with the patient, she periodically visited the outpatient clinic to support care coordination. She stated:

*“When you enter the consultation room, you naturally feel that the doctor knows best. … Even if you prepare questions in advance, once you are standing there, you hesitate. You wonder whether it is appropriate to say them.” [P16]*

Here, deference was described as a “natural” response rather than a consciously chosen strategy. This phrasing suggests that hierarchical positioning was experienced as an implicit norm embedded in the clinical setting. Across the interviews, such accounts indicated that hierarchical deference functioned as a background normative framework. Rather than being imposed solely within specific interactions, it appeared socially reinforced and culturally internalized, shaping expectations regarding when and how questioning medical judgment is considered appropriate.

### Emerging social expectations for legitimate participation in decision making

Alongside hierarchical norms, caregivers articulated emerging expectations for more inclusive and autonomous-supportive forms of decision making. These expectations were framed not as a rejection of medical expertise but as a call for broader social recognition of patient and caregiver voices. P8, a married man in his mid-forties who accompanied his mother to endocrinology visits for diabetes management approximately once every two months, described how cultural expectations surrounding authority might be shifting:

*“In the end, it is all about communication. In the drama Hospital Playlist, doctors are empathetic and devoted to their patients. But in reality, that kind of doctor does not really exist. Expecting that feels like a dream. … Still, times are changing. Maybe it is time to move beyond those socially suppressed norms. Just because someone is a patient or a caregiver does not mean they have to act in a certain way. It is our body, our family’s health. … There is no reason to feel intimidated. I think society should reach a point where patients’ and caregivers’ questions are accepted as natural.” [P8]*

In this account, the emphasis extended beyond interpersonal empathy to the social normalization of questioning. The statement that questions should be “accepted as natural” suggests a desire for a broader cultural recognition of participatory legitimacy. Similarly, P10, a married man in his late forties who accompanied his mother for diabetes-related outpatient visits every two months, articulated an ideal of decision making grounded in socially supported autonomy. Although he did not live with the patient, he regularly attended consultations to assist with understanding and coordination.

*“An ideal decision making process would create an environment where patients can truly make decisions. … Socially, there needs to be more emphasis on personal agency. Instead of relying on authority figures, patients should be able to speak up and act. … That is how autonomy can be guaranteed in the decision making process. In that process, caregivers can serve as an important bridge between the patient and the doctor. But first, I think our awareness needs to change.” [P10]*

In this account, SDM was framed as being dependent on broader societal shifts in awareness and normative expectations. The emphasis on changing “awareness” suggests that SDM was understood not merely as a clinical technique but as contingent upon collective cultural transformation. Caregivers’ accounts reflected the coexistence of established hierarchical norms and evolving expectations of participatory legitimacy. Although medical authority continued to be recognized as socially authoritative, participants simultaneously articulated a vision in which questioning, dialogue, and caregiver mediation were culturally normalized rather than restrained. These patterns underscore how societal norms shape both the constraints and emerging possibilities of SDM.

## Discussion

This study examined how family caregivers of patients with chronic illnesses in Korea experience and interpret their participation in SDM at multiple ecological levels. Using SEM as an interpretive framework, the findings suggest that caregiver participation may not be adequately captured as an individual disposition or a purely interpersonal behavior. Instead, caregiver involvement appeared to take shape through interacting influences at the individual, interpersonal, organizational, and societal levels [35,36]. This multilevel interpretation is consistent with qualitative implementation research indicating that structural and organizational arrangements can substantially condition how SDM principles are enacted in routine practice [26,49]. This also aligns with qualitative studies showing that caregiver participation varies by relational positioning and care context rather than by operating as a uniform or stable role [48,50–52].

At the individual level, caregivers frequently described decision-related uncertainty accompanied by emotional strain, especially in situations perceived as clinically complex, urgent, or difficult to anticipate. Previous studies have emphasized that chronic illness management involves evolving trajectories with repeated monitoring and treatment adjustments rather than isolated choice points [2,3]. Research has also suggested that uncertainty may intensify cognitive and emotional burden in chronic care [6,7]. Consistently, the caregivers in this study made sustained efforts to seek, interpret, and verify information. Importantly, their accounts often framed these efforts less as empowerment per se and more as responses to perceived vulnerability and responsibility within family-centered care. In the Korean context, where family responsibility and filial obligations remain socially salient [9,10], information seeking appears to function as a way to reduce perceived risk to family members and manage anxiety regarding misunderstanding decision-relevant details. Therefore, caregiver engagement may be shaped not only by activation or health literacy but also by culturally patterned accountability embedded in family roles.

At the interpersonal level, caregiver participation was influenced by interactional conditions within clinical encounters and perceived asymmetries in authority and knowledge [53,54]. Even where SDM is normatively emphasized, prior studies have suggested that hierarchical communication patterns may persist in ways that restrain reciprocal dialogue [12,28,55]. In this study, caregivers described brief consultations, routinized exchanges, and relational cues that shaped their judgments about when and how to raise questions [25,27]. These accounts reflect what was conceptualized as a participation paradox, which means that caregivers were implicitly expected to coordinate care and support decisions without consistently experiencing structured opportunities for clarification or deliberation [8,14]. This hesitation was attributed to multiple sources. Some participants emphasized authoritative atmospheres or time pressure within encounters, whereas others described internalized expectations regarding appropriate role conduct, including norms of deference and filial responsibility [9,12]. This co-occurrence suggests that interpersonal participation may be shaped simultaneously by encounter-level dynamics and broader normative expectations.

At the organizational level, caregiver experiences highlighted how service delivery structures within hospital systems condition SDM engagement [27,48]. Differences in institutional scale, scheduling systems, referral pathways, and diagnostic infrastructure were described as influencing communication and perceived continuity of care [52]. Prior research has noted that endorsement of patient-centered or family-centered care does not automatically translate into routinized mechanisms for caregiver inclusion [17,27]. In the present study, caregivers described assuming responsibility for synthesizing fragmented information, navigating appointment systems, and translating standardized guidance into patient-specific decisions across care settings [5,8]. These patterns suggest a mechanism of structural externalization, whereby coordination and interpretive burdens may shift to families, although SDM remains formally endorsed. Such organizational conditions may constrain participation in ways that are not readily addressed by individual motivation or communication strategies alone [26,27].

At the societal level, the findings indicated that caregiver participation was shaped by three interrelated mechanisms derived from participants’ accounts: culturally internalized hierarchical deference to medical authority, socially normalized expectations of familial responsibility, and evolving normative shifts toward participatory legitimacy. Caregivers frequently described physicians as occupying socially elevated and epistemically privileged positions, consistent with prior analyses of professional authority in Korean medicine [11,53]. Hesitation in questioning was linked not only to interactional constraints but also to socially embedded expectations regarding appropriate role conduct. Although previous studies have documented hierarchical norms and filial responsibility in East Asian healthcare settings [12,28], the present findings illustrate how these norms were interpreted and negotiated in caregivers’ everyday experiences. At the same time, the participants articulated their expectations that questioning and dialogue should be recognized as legitimate components of care. Rather than indicating a linear shift from hierarchy to autonomy, the empirical material suggests a layered coexistence between deference and emerging participatory aspirations.

At different levels, caregiver involvement may be understood as a contextually adaptive response to intersecting institutional, relational, and normative conditions [51]. Caregivers described functioning as mediators facilitating exchanges between patients and clinicians, navigators coordinating institutional pathways, and interpreters translating medical information or representing patients’ perspectives when direct participation was limited. Although similar functional roles have been noted in other clinical contexts [8,14], this study situates these roles within an integrated multilevel framework that focuses on the interplay between sociocultural norms and healthcare structures. Importantly, caregivers were rarely positioned as autonomous decision makers; rather, their participation appeared to be oriented toward facilitating patient engagement within existing constraints.

Collectively, these findings contribute to the understanding of SDM in several ways. First, by applying a social-ecological framework, the study situates caregiver participation within interacting structural and cultural layers rather than treating it solely as an individual-level phenomenon [35,36,48]. Second, it extends the research on SDM in East Asian contexts by analytically highlighting caregivers as distinct actors whose roles may be simultaneously expected and constrained [8,12]. Whereas much prior SDM research has focused primarily on physician–patient dyads, the present analysis highlights the caregiver as a meso-level mediator embedded within family-centered systems. Third, it identifies the mechanisms observed in this study—the hierarchical deference, normative family responsibility, participation paradox, and structural externalization—that may help explain the variability in SDM enactment across institutional and sociocultural settings. These contributions are interpretive and grounded in the qualitative data of this study and should be considered within the context of this study.

The study findings also suggest that supporting caregiver-inclusive SDM may require attention to conditions operating at multiple levels [26,36]. At the individual level, interventions acknowledging uncertainty and informational strain may complement strategies aimed at strengthening decisional confidence [3,34]. At the interpersonal level, communication approaches that explicitly address interactional norms and asymmetries may facilitate more reciprocal dialogue [25,56]. At the organizational level, clearer caregiver inclusion protocols and structured information pathways may reduce the coordination burdens described by the participants [27,48]. At the societal level, broader discourse and policy initiatives that legitimize caregiver questioning may gradually influence normative expectations [12,18]. Alignment across levels rather than changing at a single point may enhance the practical feasibility of SDM in family-centered contexts.

This study has several strengths. By employing a social-ecological framework, the study examined caregiver participation across interaction levels, individual, interpersonal, organizational, and societal, thereby clarifying how interactional experiences are embedded within broader institutional and sociocultural conditions. Focusing on caregivers of patients with chronic illnesses in Korea allowed for a contextually grounded analysis of SDM in a healthcare system in which family involvement remains structurally integrated into care processes. This approach provided a more comprehensive account of participation dynamics that extend beyond individual motivation or communication behavior.

This study has some limitations. As a qualitative study conducted within a single national setting, the findings are context-specific and not intended for statistical generalization. The sample consisted exclusively of the family caregivers of the patients with chronic illnesses, which may limit their applicability to other caregiving configurations or clinical contexts. Because each disease-specific focus group comprised only five caregivers, the design did not support an in-depth, comparative analysis of disease-specific decision making dynamics; thematic sufficiency was therefore assessed at the cross-disease level, and condition-specific nuances may not have been fully captured. In addition, participants were recruited through a Seoul-based research agency and were, on average, relatively highly educated, so the findings reflect the experiences of a comparatively educated, urban caregiver group rather than the broader Korean caregiving population, and their transferability to rural, less-educated, or differently recruited caregivers may be limited.

Participant narratives represented subjective interpretations that may vary across institutions and regions. Additionally, the cross-sectional design did not capture potential changes in caregiver roles over time, and the absence of patient and clinician perspectives restricted insights into how interactional dynamics are experienced and interpreted by different actors within the clinical encounter.

Future research can extend these findings in several directions. Studies across additional clinical contexts, such as pediatric chronic illness, progressive cognitive decline, and end-of-life decision making [14,16], may clarify how caregiver roles vary when decisional capacity, dependency, and normative responsibility differ. Including diverse caregiver types, such as spouses or nonfamilial caregivers, would further illuminate how relational positioning shapes participation. Longitudinal designs may help trace how caregiver involvement evolves across illness trajectories and care transitions and whether sustained inclusion is associated with patient or caregiver outcomes. Intervention-oriented research, including the pilot testing of structured caregiver consultation protocols or organizational models that formalize caregiver information pathways, can assess how institutional support influences SDM implementation in family-centered settings. Finally, comparative research across sociocultural and institutional contexts may refine SDM frameworks by identifying how hierarchical norms, family responsibility expectations, and participatory legitimacy operate in different healthcare systems.

## Conclusion

This study examined how family caregivers of patients with chronic illnesses in Korea experience and interpret their participation in SDM at individual, interpersonal, organizational, and societal levels. These findings indicate that caregiver involvement extends beyond passive accompaniment and is shaped through emotional, relational, structural, and sociocultural interactions. Caregiver participation emerged as overlapping functional contributions, including mediating communication, navigating healthcare systems, and interpreting medical information, rather than as a single, fixed role. Importantly, caregivers were not positioned as autonomous decision makers; rather, their accounts reflected efforts to facilitate patient engagement within existing hierarchical, organizational, and family-centered contexts. By situating caregiver participation within a social-ecological framework, this study highlights that SDM enactment in family-centered healthcare environments is influenced not only by individual communication behavior but also by institutional design and culturally embedded norms. Recognizing caregivers as facilitators who support patient engagement while maintaining patient values and preferences as central to decision making may strengthen the practical implementation of SDM in chronic illness care.

## Supporting information

S1 File Interview guide (English translation). (DOCX)

S2 File Codebook summary. (DOCX)

S3 File Additional anonymized excerpts supporting thematic findings. (DOCX)

S4 File COREQ checklist. (PDF)
