# Supplementary material for: Whose voice counts? Family caregivers’ roles in shared decision making for chronic illness in urban South Korea: A qualitative study
Source: PLoS One. 2026 Aug 6;21(8):e0353308. doi: 10.1371/journal.pone.0353308 (PMC13446666; doi:10.1371/journal.pone.0353308)
Supplement: Supplementary file 2 [file pone.0353308.s001.docx]

**S1 File. Interview guide.**

**Whose Voice Counts? Family Caregivers' Roles in Shared Decision Making for Chronic Illness in Urban South Korea: A Qualitative Study**

**Methodological transparency note.**

This file is provided as de-identified supporting information to improve transparency about the focus-group interview procedures. The guide documents the core domains used across the four disease-homogeneous focus groups and was applied flexibly rather than as a fixed survey instrument.

The guide was not used as a deductive Social-Ecological Model coding template. Interviews were conducted first, transcripts were analyzed inductively, and SEM was applied only after theme development as an interpretive framework. Questions therefore elicited caregivers' accounts of communication, information seeking, and decision-related participation rather than forcing responses into predetermined SEM levels.

Because the study analyzes caregiver perspectives, the questions were designed to explore caregivers' experiences and interpretations. They were not intended to reconstruct patient preferences, clinician intentions, or institutional policy directly.

**Note.**

This semi-structured guide for focus-group interviews with family caregivers was developed from the shared decision making literature and pilot-tested with caregivers who were not included in the main study. Interviews were conducted in Korean; the guide is presented here in English translation. The questions served as a flexible framework, and the moderator adapted the wording, order, and follow-up probes to the flow of each discussion. The thematic groupings below (understanding the illness; talking with providers; using the hospital; the wider social context) organized topic coverage during the interviews. Although these domains broadly anticipate multiple ecological levels, they were used only to ensure breadth of discussion and were not treated as analytic categories: the codes and their later organization under the Social-Ecological Model were developed inductively during analysis (see S2 File and Methods). The SEM was applied post hoc as an interpretive framework, not as a deductive coding structure.

Some questions used frequency or scaling language as prompts to facilitate discussion, but these responses were not treated as quantitative measures; rather, they were followed up qualitatively to elicit caregivers’ narratives, meanings, and contextual explanations.

**Opening question**

1. Among the doctors or nurses you have met recently, who is the most memorable, and why?

**Understanding the illness and managing information**

2. How well do you understand the patient's health condition (time of diagnosis, frequency of hospital visits, precautions)?

3. How did you feel when your family member was first diagnosed with the current chronic illness? What went through your mind?

4. Since the diagnosis, where do you mainly obtain information about the treatment?

5. As a caregiver, how do you feel when you accompany the patient to the hospital? What has your experience as a caregiver been like?

6. How interested are you in the patient's medical decisions? Please describe any experience of decision making related to the treatment process while accompanying the patient.

7. How do you assess whether the patient understands the medical information? If not well, what do you think are the reasons?

**Talking with providers**

8. How often do you talk with the patient about the chronic illness?

9. How often do you accompany the patient for regular treatment?

10. If you accompanied the patient at the first diagnosis, how was the communication with the physician?

11. How much do you trust the physician? How well do you feel you know the physician?

12. For ongoing treatment, how, how much, and how sufficiently did the medical staff (physicians, nurses) explain things? Were there aspects of the communication that were helpful or difficult?

13. Did you receive explanations about medical terms, tests, procedures, surgery, or patient education?

14. Do you think the patient had sufficient options (treatment choices) in their own decision? How large do you think the patient's “share” in the decision was?

15. If decision making authority were rated from 0 to 10, how much did the patient hold? Who held the rest, and how much?

16. Apart from the actual situation, what distribution of decision making authority do you think would be ideal?

17. What do you do when your opinion and the patient's opinion conflict?

18. What do you do when the patient's and the medical staff's opinions conflict, and how do you mediate?

**Using the hospital**

19. How often do you go to the hospital for regular treatment, and what goes through your mind when you go?

20. How long does a single hospital visit usually take?

21. How much disease-related information do you receive at the hospital?

22. Do you feel that the hospital sufficiently explains the process of making appointments and receiving treatment?

23. What is difficult for you as a caregiver at the hospital, and what kind of support would be helpful?

**The wider social context**

24. What do you think makes a good doctor?

25. What do you think makes a good nurse?

26. What do you think makes a good patient?

27. What do you think makes a good caregiver?

28. What are your thoughts on social and governmental support for patients with chronic illness and their caregivers?

**Closing**

29. What could be improved so that patients and physicians exchange views more actively and patients participate more proactively in decisions?

30. What is the most difficult part of talking with physicians or other medical staff?

31. What do you hope for from physicians, other medical staff, and hospitals?

32. Is there anything you were not able to say that you would like to add?
