# Supplementary material for: Whose voice counts? Family caregivers’ roles in shared decision making for chronic illness in urban South Korea: A qualitative study
Source: PLoS One. 2026 Aug 6;21(8):e0353308. doi: 10.1371/journal.pone.0353308 (PMC13446666; doi:10.1371/journal.pone.0353308)
Supplement: Supplementary file 3 [file pone.0353308.s002.docx]

**S2 File. Codebook summary.**

***Whose Voice Counts? Family Caregivers’ Roles in Shared Decision Making for Chronic Illness in Urban South Korea: A Qualitative Study***

**Analytic transparency note.**

This codebook summary is provided to clarify how the analytic process moved from inductive coding to post hoc SEM interpretation. It is a de-identified summary rather than the full working codebook or a transcript release, because the full transcripts contain potentially identifying information and are restricted under the Korea University IRB approval.

Disease group was retained as a contextual identifier during coding, but disease category was not treated as an explanatory variable for subgroup comparison. This reflects the manuscript's aim: to examine cross-cutting multilevel conditions shaping caregiver involvement across chronic illness contexts.

SEM grouping was applied after inductive coding and team discussion. Codes were not discarded or reassigned solely to fit SEM levels; where a code crossed levels, it was placed according to its primary analytic function, and cross-cutting material was retained as such.

Description. This document summarizes the inductive coding framework applied to the focus-group transcripts using MAXQDA 24. First-cycle codes were generated from caregivers' accounts through open coding, and the codebook was revised through seven iterations as code definitions were clarified, overlapping codes were merged, and new codes could still be added when warranted by the transcripts. Only after inductive coding and theme development were completed were the resulting primary codes placed in the post hoc interpretive grouping shown in the first column. This grouping follows the Social-Ecological Model to help readers compare the supplementary codebook with the organization of the revised Results; it was not used to generate the original codes or to force data into predefined levels. To protect participant confidentiality in accordance with the approval of the Institutional Review Board of Korea University (KUIRB-2024-0156-04), the table reports code definitions and de-identified, paraphrased example indicators rather than verbatim transcript excerpts; no information that could identify individual participants or institutions is included. A small number of inductively generated codes (for example, 4.4 Impact of COVID-19) did not develop into foregrounded themes in the final SEM-organized analysis and are retained here for completeness and transparency about the full coding process.

| Post hoc interpretive grouping | **Primary code** | **Definition** | **Representative indicators (de-identified)** |
| --- | --- | --- | --- |
| Individual-level grouping | 1.1 Illness-related characteristics | Features of the chronic illness as described by caregivers, including onset, symptoms and signs, disease course over time, complications, treatment, and prognosis. | Time of diagnosis; family history; tests / procedures / consultations; health management (medication, diet, exercise); everyday normalization of symptoms in ageing and chronic illness. |
|  | 1.2 Information-seeking behavior | Caregivers’ actions to access and select the information they need. | People (family, acquaintances, providers); internet (online patient communities, video platforms, TV); other sources (articles, pamphlets). |
|  | 1.3 Patient health status | Caregivers’ accounts of the patient’s past and current health status. | Past (e.g., hospitalization, changes before/after dialysis); present (e.g., difficulty with self-management). |
|  | 1.4 Patient emotions | Emotions the patient experiences while managing and being treated for the illness. | Hesitation; fear; regret; stress; disappointment; relief; heightened sensitivity. |
|  | 1.5 Health literacy | Ability to understand and use medical information to make health-related decisions. | Positive (active information seeking); negative (difficulty with technical terms, inconsistent information, knowledge asymmetry, low receptivity); neutral (self-assessment). |
|  | 1.6 Absence of a caregiver | Situations in which a caregiver was absent (e.g., at the time of diagnosis) and the implications for care and decisions. | Concerns related to managing treatment alone; how absence shaped a treatment choice. |
|  | 1.7 Age-related characteristics | Features of illness management associated with the patient’s age. | Constraints on treatment options for older patients (e.g., limited mobility). |
| Interpersonal-level grouping | 2.1 Communication with physicians | Process of exchanging medical information (health problems, symptoms, treatment) between caregiver/patient and physician. | Positive (recommendation, persuasion); negative (communication difficulty, perceived inattention, defensive communication, reduced trust); neutral (general perceptions/experience). |
|  | 2.2 Communication with nurses | Process of exchanging medical information between caregiver/patient and nurses. | Positive (constructive communication, nurses’ information-relay role); negative; neutral. |
|  | 2.3 Patient’s communication | The patient’s own exchange of medical information with caregivers and providers. | Positive (patient-initiated communication); negative (patient communicates differently to caregiver vs. physician; voicing dissatisfaction to the caregiver). |
|  | 2.4 Communication with non-physician staff (administration, laboratory) | Interactions with allied and administrative personnel who support the patient’s care. | Navigating administrative and laboratory contacts; information hand-offs across roles. |
|  | 2.5 Caregiver’s role | The caregiver as a companion supporting the therapeutic relationship between providers and patient. | Relaying information and aiding comprehension; supporting the patient’s expression; emotional support; gathering information and asking questions; partner in the decision making process. |
|  | 2.6 Provider information provision | Information that providers do or do not give to the caregiver/patient. | Information given (e.g., medication guidance); information not given (e.g., insufficient pre-procedure or pre-test explanation). |
|  | 2.7 Power relations / experienced SDM | Perceived power imbalance between providers and caregiver/patient, and accounts of shared decision making as actually experienced. | Hierarchical relationship; information asymmetry; perceived share of decision making authority. |
|  | 2.8 Trust–distrust relationship | Trust or distrust arising between providers and the caregiver/patient. | Reliance on a trusted provider; erosion of trust following negative experiences. |
|  | 2.9 Alignment–conflict relationship | Alignment or conflict among providers, caregivers, and patients. | Cooperative alignment across dyads (physician–caregiver, patient–caregiver, etc.); interpersonal conflict. |
| Organizational-level grouping | 3.1 Characteristics of tertiary hospitals | Features of tertiary (advanced general) hospitals that provide specialized care for severe conditions. | Positive (comprehensive range of departments); negative (time-pressured consultations, limited inter-department coordination, complex processes, repeated tests). |
|  | 3.2 Characteristics of secondary hospitals | Features of general hospitals (secondary care). | Access and travel considerations; scope of services. |
|  | 3.3 Characteristics of primary care clinics | Features of local clinics focused on outpatient care for less severe conditions. | Positive (approachability, more time, service orientation); negative (difficulty declining recommended tests or medications). |
|  | 3.5 Inter-institutional comparison | Caregivers’ comparisons across hospital tiers and individual institutions. | Tertiary vs. general; general vs. local clinic; tertiary vs. local clinic; comparisons between individual hospitals. |
|  | 3.6 Information provision by the hospital | Whether the hospital, as an organization, provides information to caregivers/patients. | Information provided (e.g., pamphlets, cross-checking); information lacking (e.g., abundant at admission but sparse at discharge; variability across hospitals). |
|  | 3.7 Accountability and uncertainty of hospital care | Medicine as an essential public service together with the structural difficulty of clearly establishing medical error. | Structural difficulty of demonstrating harm or disadvantage to the patient. |
| Societal-level grouping | 4.1 Shared decision making (caregiver, patient, provider) | Caregivers’ implicit and ideal conceptions of shared decision making across actors. | Role of physician, nurse, caregiver, and patient; burden of holding decision authority; passive patient decision making. |
|  | 4.2 Economic factors | Economic circumstances of the caregiver/patient within the care process. | Financial difficulty associated with treatment. |
|  | 4.3 Ideal image of the physician | Caregivers’ and patients’ conceptions of an ideal physician. | Attributes attributed to a “good” physician. |
|  | 4.4 Impact of COVID-19 | Features arising in care after the COVID-19 pandemic. | Changes in communication and access following the pandemic. |
|  | 4.5 Particularities of the Korean medical system | Distinctive structural features of the Korean hospital system. | Concentration of technology in and reliance on tertiary hospitals; structural barriers to using local clinics; status differences among professional roles. |
| Cross-cutting grouping: suggested improvements | 5.1–5.5 Improvements proposed by participants | Caregivers’ suggestions for improving care and decision making (coded across levels). | Information (clearer explanation, patient education, online education); communication (patient-tailored, empathetic communication); system/technology (consultation manuals, more efficient waiting/queue systems); government policy (workforce, decentralization of facilities); other improvements. |

**Note.**

Code numbering follows the working codebook; minor gaps in numbering reflect codes that were merged or removed during the seven rounds of revision. The first column is a reporting and navigation aid for readers, not a record of the first-cycle coding sequence. “Positive / negative / neutral” sub-codes denote the valence of the caregiver’s account, not an evaluative judgment by the research team. Codes describing the patient (for example, 1.3 Patient health status, 1.4 Patient emotions, and 2.3 Patient's communication) were applied to caregivers' accounts and therefore represent the patient as reported by the caregiver, consistent with the caregiver-perspective design of the study.

Mapping note. Where a code could plausibly relate to more than one interpretive level, the team assigned it to the grouping that best reflected its primary analytic function in the revised manuscript. Participants' suggested improvements were retained as a cross-cutting category rather than forced into a single SEM level.
