# Supplementary material for: Whose voice counts? Family caregivers’ roles in shared decision making for chronic illness in urban South Korea: A qualitative study
Source: PLoS One. 2026 Aug 6;21(8):e0353308. doi: 10.1371/journal.pone.0353308 (PMC13446666; doi:10.1371/journal.pone.0353308)
Supplement: Supplementary file 4 [file pone.0353308.s003.docx]

**S3 File. Additional anonymized interview excerpts.**

**Whose Voice Counts? Family Caregivers' Roles in Shared Decision Making for Chronic Illness in Urban South Korea: A Qualitative Study**

**Selection, anonymization, and interpretation note.**

This file supplements the quotations in the main manuscript with additional anonymized excerpts. The excerpts were selected to illustrate how the manuscript's interpretations are grounded in caregiver accounts at individual, interpersonal, organizational, and societal levels; none should be read as direct evidence of clinician intent, patient preference, institutional policy, or population-level dynamics.

The excerpts were translated from Korean through the consensus-based translation and back-translation process described in the manuscript. Names, institutions, locations, and potentially identifying clinical details were removed or generalized in accordance with the Korea University IRB restriction on public transcript release.

The excerpt labels are internal de-identified labels for this supporting file only and are not mapped to the participant identifiers used in the main manuscript, to reduce re-identification risk.

**Description.**

This file provides additional de-identified excerpts from the four focus-group interviews (FGIs), presented so that readers can assess the link between the data and the interpretations reported in the main text. None of the excerpts below appears in the main manuscript; together with the in-text quotations they illustrate the range of caregiver accounts supporting each theme. Excerpts are organized under the four levels of the Social-Ecological Model (SEM) that was applied post hoc as an interpretive framework during analysis.

All excerpts were translated from Korean following the consensus-based translation and back-translation procedure described in the Methods. To protect participant confidentiality in accordance with the approval of the Institutional Review Board of Korea University (KUIRB-2024-0156-04), names, institution names, and other potentially identifying details have been removed or generalized (for example, specific hospitals are rendered as “a tertiary hospital,” “a general hospital,” or “a local clinic”). Participants are labeled with within-file codes by disease group (HTN = hypertension; DM = diabetes; HD = hemodialysis; HEART = Heart disease; e.g., HTN-01). These codes are internal to this file and are deliberately not mapped to the participant numbering (P1–P20) used in the main text, so that excerpts in the manuscript and in this file cannot be cross-linked to re-identify individuals. Ellipses (…) indicate omitted speech; square brackets mark generalized or clarifying text.

**Individual level: internalized responsibility, information-seeking, and health literacy**

“After my mother started the medication, her hands and feet suddenly turned very cold. When we went back the next month to refill the prescription and I asked whether this was a different symptom, [the doctor] said the blood-pressure medication can do that. If they had explained this beforehand it would have been so much better, because for that whole month until the next visit I was anxious, wondering “why is this happening?” … So now I look things up in [online] patient communities and search the drug names in advance. That habit comes from that experience.”

*— HTN-03 (daughter; mother with hypertension)*

“When a prescription comes out there is the medication leaflet, and then I go into [a web portal’s] knowledge service and look it up—what the ingredients are, what the side effects are. Then I tell my mother, “Take this, and if you feel dizzy or nauseous tell me, because we will need to change it quickly,” since older people like my mother, who is over seventy, do not have the expert knowledge to check for themselves. … And honestly I also use fear: “Mom, if you don’t take it something terrible will happen, I’ll collapse.” The pharmacist does not explain the side effects either, so it is something the children or caregivers have no choice but to handle.”

*— DM-05 (daughter; mother with diabetes)*

“At the hospital they just hand you a booklet when dialysis starts, and the booklet only lists what you cannot eat—this and that—it does not tell you concretely what you can eat. That was frustrating. When I am next to my mother saying “you can’t have this, you can’t have that,” she always says, “then what am I supposed to eat?” and I have nothing to say, so a lot of conflict arises. In the end I got most of my information from the [online] café, or from other caregivers waiting during dialysis.”

*— HD-02 (daughter; mother on hemodialysis)*

“When I am given a prescription I look the drug up myself—these days the side effects and so on are described in detail online—so I read through it at least once, every time. The hospital does not explain side effects, so I check on my own.”

*— HTN-02 (adult child; father with hypertension)*

“The doctor had been telling us for a few years that she needed dialysis, but we were scared—we did not even know what dialysis was, and we had heard it is something you do for life and that it is hard—so my mother said she could not do it and we kept putting it off. The doctor urged us at least to have the access created in her arm in advance in case of an emergency, so we did that much. Then she actually collapsed and went to the ER, and because the access was already there they could start dialysis through it. Afterward I thought we should have listened to the doctor and done it earlier.”

*— HD-03 (adult child; mother on hemodialysis)*

“I am the youngest by a lot, and my father is now eighty-five, so my older brother takes the lead and I tend to hear things second-hand from him. My father managed his own diet and medication for over twenty years, but once he passed eighty the control slipped, so he has to follow the doctor’s word more. His confidence in managing it himself is fading year by year, and before long my brother will probably have to make the decisions.”

*— DM-03 (son; father with diabetes)*

**Interpersonal level: relational constraint, deference, and patient positioning**

“You end up lowering yourself. When you ask something, you find yourself prefacing it with “Sorry, but…” before the question.”

*— HTN-04 (son; parent with hypertension)*

“After the consultation my mother was unhappy and muttering in the waiting room, so I told her to be quiet—worried it might be overheard and count against us. Afterward I felt guilty, because I should have understood her feelings first; instead, fearing she might be disadvantaged, I was the one telling her to stop.”

*— HTN-03 (daughter; mother with hypertension)*

“My mother tells the doctor something different from what she tells me. To me she says she was short of breath or that her chest felt tight, but in front of the doctor she says everything was fine — in this instance she also concealed that she had not been taking her medication well and that she was still smoking, so I kept correcting it: “No, she only took it at first and then stopped; and she is still smoking.” She also refused the stent procedure because the groin insertion was painful, even though the hospital said she could die without it. So I pressed her, and I asked the doctor whether it could be done another way; in the end they inserted it through the arm instead of the groin, and she was more satisfied because it hurt less.”

*— HEART-01 (daughter; mother with cardiac disease)*

“For the vascular-access surgery they normally use the non-dominant arm, but my mother is left-handed and they did it on her left arm. I asked them not to, but the doctor said the vessels were better on the left. In the end that access kept getting blocked—she had it re-done more than five times. When we later went to other hospitals they asked why it had been placed on the dominant side, and why a graft had not been suggested given how poor the vessels were. If it had been suggested my mother would have considered it, but because the doctor did not raise it she did not ask—patients who simply do as they are told assume there must be a reason. It cost her almost a year of suffering, and that is why we eventually changed hospitals.”

*— HD-04 (daughter; mother on hemodialysis)*

“The doctor barely explains the medications, so I just look them up on the internet and know roughly what they are for. It is hard to meet the doctor as a caregiver, and we only go once or twice a year. When my mother asks things, older people ramble and the doctors clearly find it tiresome, so I only ask the strictly necessary questions—just the things we absolutely need to know.”

*— HEART-04 (son; mother with cardiac disease)*

**Organizational level: institutional scale, inter-institutional differences, and information provision**

“At the major tertiary hospital, even if you want to ask, the nurses and doctors do not give you the chance—they say what they have to say, “take this, do that,” and they are already leaving while you are still looking at them, because they are so busy. At the general hospital it is much easier to ask; when the nurse comes out to the clinic area you can go up and ask first. And with appointments, at the tertiary hospital if you say you really cannot make a date it is automatically pushed three months later, whereas the general hospital is far more flexible, sometimes even offering an afternoon slot the same day.”

*— DM-01 (daughter; mother with diabetes)*

“The special co-payment reduction for the pre-dialysis vascular procedure is applied differently from hospital to hospital. At the previous hospital it was covered regardless of the dialysis date because it is a preparatory procedure; at the hospital I switched to, they said it is only covered if dialysis is performed that same day. My mother’s vascular procedure did not go well and was redone more than five times, so the cost matters enormously—but there is no consistent standard, and the patient, already exhausted, has to explain it all over again each time.”

*— HD-04 (daughter; mother on hemodialysis)*

“What the hospital gives is not really information—it is just a thin booklet, and they hold a kidney-disease prevention lecture, but the notes I compiled myself from the [online] café over several months were far more useful, so I never once asked the hospital for materials. Years ago there were almost no renal-diet products available here, so I searched in [another country] where there were more kidney patients, found products such as low-protein noodles, and imported them myself, dealing with customs each time.”

*— HD-05 (wife; husband on hemodialysis)*

**Societal level: medical authority, hierarchical norms, and emerging expectations**

“At the hospital you are not even the “second party.” It feels more like being at the very bottom of the hierarchy. The second party can at least talk back once, but in the hospital you are near the bottom—and if you raise your voice there, even the other patients around you look at you strangely, as if to say, “what is wrong with that person?””

*— HTN-01 (son; parent with hypertension)*

“If I treat the doctor as a god, then he is the one who has to cure the illness, so I am in the position of someone who must hold him up unconditionally—someone who cannot dare to talk back.”

*— HTN-05 (son; mother with hypertension)*

“First of all, I am the sick one—the weaker party. I cannot help but be the weaker party, because the doctor is the one who has to treat us. … So the doctor takes about 80–90% of the initiative, and the remaining 10% or so is just me asking what I am curious about.”

*— DM-05 (daughter; mother with diabetes)*

“My view is that even though medicine is a profession, it is like shopping—if I am willing to pay the highest price, I can choose the best providers. So for a given condition I select the top specialists, book them all even if it takes a year, meet each one, and assess the doctor’s character through the consultation—how authoritarian they are, how well they counsel—before deciding. Because my parents are elderly and cannot explain their own symptoms or ask the right questions, I attend the consultations myself and ask a great many questions.”

*— HEART-03 (daughter; parents with cardiac and other chronic conditions)*
